# The Ready Reference Handbook of Diseases of the Skin

**Published:** 1905-10

**Authors:** 


**The Ready Reference Handbook of Diseases of the Skin.** By **George Thomas Jackson,** M.D., Chief of Clinic and Instructor in Dermatology, College of Physicians and Surgeons (Columbia University), New York. Fifth edition, enlarged and revised. Duodecimo, 676 pages, with 91 engravings and 3 colored plates. Philadelphia and New York: Lea Brothers & Co. 1905. (Cloth, $2.75 net.)

That the fifth edition of Dr. Jackson's work on diseases of the skin should be so soon called for is not only flattering to the author, but proves that the profession can only be satisfied with the very best works of this nature.

The work in this edition has been subjected to careful revision and new conditions have received special consideration. The present volume more than justifies expectations and realises the wish of the writer to present a practical, up to date compact treatise. The alphabetical arrangement of diseases of the skin is to be commended as simplifying the book of the student ministering to the convenience of the practitioner.

This volume as a whole stands in the front rank of treatises of this special subject. Dr. Jackson is to be congratulated upon the plain and straight forward style which he adopts and the absence of all affectation which characterises his works.

